# Reaching the limits of antipsychotic treatment: the upper end of severe schizophrenia in forensic institutions - a case report

**DOI:** 10.1192/j.eurpsy.2024.1213

**Published:** 2024-08-27

**Authors:** V. Watzal, T. Stompe

**Affiliations:** ^1^Department of Psychiatry and Psychotherapy; ^2^Comprehensive Center for Clinical Neurosciences and Mental Health, Medical University of Vienna, Vienna; ^3^Forensic therapeutic center Goellersdorf, Goellersdorf, Austria

## Abstract

**Introduction:**

Severe schizophrenia is often closely related to delinquency resulting in relative overrepresentation of these manifestations of disease in forensic institutions.

**Objectives:**

The aim of the present work is to report the therapeutic challenges in a case of severe schizophrenia in a forensic institution from a clinical viewpoint as a basis for discussion.

**Methods:**

The case report is based on the available clinical documentation, exploratory interviews as well as a structured clinical interview (PANSS).

**Results:**

Presenting a case of a 41-year-old, male Caucasian inpatient suffering from a catatonic schizophrenia, we report the challenges in treatment of chronic, major schizophrenic disease resistant to antipsychotic medication. Without any previous criminal convictions, he has been instutionalized in a forensic psychiatry after a bodily harm to a random stranger about three years ago. Regarding medical history, information is limited to a few inpatient admissions prior to detention documenting intravenous opioid and cocaine abuse. Initially, the patient presented sexual disinhibition and ongoing endangerment of others with frequent assaults to other patients and prison guards. From a psychopathological viewpoint several phenomenona such as delusional intuition, acoustic, tactile and coenaesthetic hallucinations, echolalia, mannerisms and thought diffusions reflect the severe course of the disease (PANSS: P 34/49, N 38/49, G 73/112; total 145/210). Therapeutic attempts with an antipsychotic combination of risperidone, olanzapine and quetiapine as well as valproic acid resulted in insufficient recovery with persistent physical assaults and florid psychosis. In reaction to that zuclopenthixol for impulse control was added. As from the beginning of this year a switch of medication by gradually replacing risperidone and zuclopenthixol with haloperidol and clozapine showed modest success. Under the current medication and therapeutic drug levels the patient does not pose endangerment to others. However, regular tonic-eye fits require supplementary treatment with biperiden, and the patient still presents frequent periods of self-harm punching himself, verbal lack of impulse control and the psychopathological phenomenona described before. In addition to pharmacological treatment the patient receives psychotherapeutic one-on-one conversations. Despite approaching all limits of the available antipsychotic repertoire, psychopathology is only insufficiently controlled leading considerations to electroconvulsive therapy as a treatment of last resort.

**Conclusions:**

Certainly, the present case is exemplary for a severely ill population of patients reaching – after a long and untreated course of disease - a chronic stage that does not sufficiently respond to a multitude of treatment attempts despite proper compliance raising the urgent need for further treatment options.

**Disclosure of Interest:**

None Declared

